# Supplementary figures and images for: A novel Oprm1-Cre mouse maintains endogenous expression, function and enables detailed molecular characterization of μ-opioid receptor cells
Source: PLoS One. 2022 Dec 19;17(12):e0270317. doi: 10.1371/journal.pone.0270317 (PMC9762562; doi:10.1371/journal.pone.0270317)

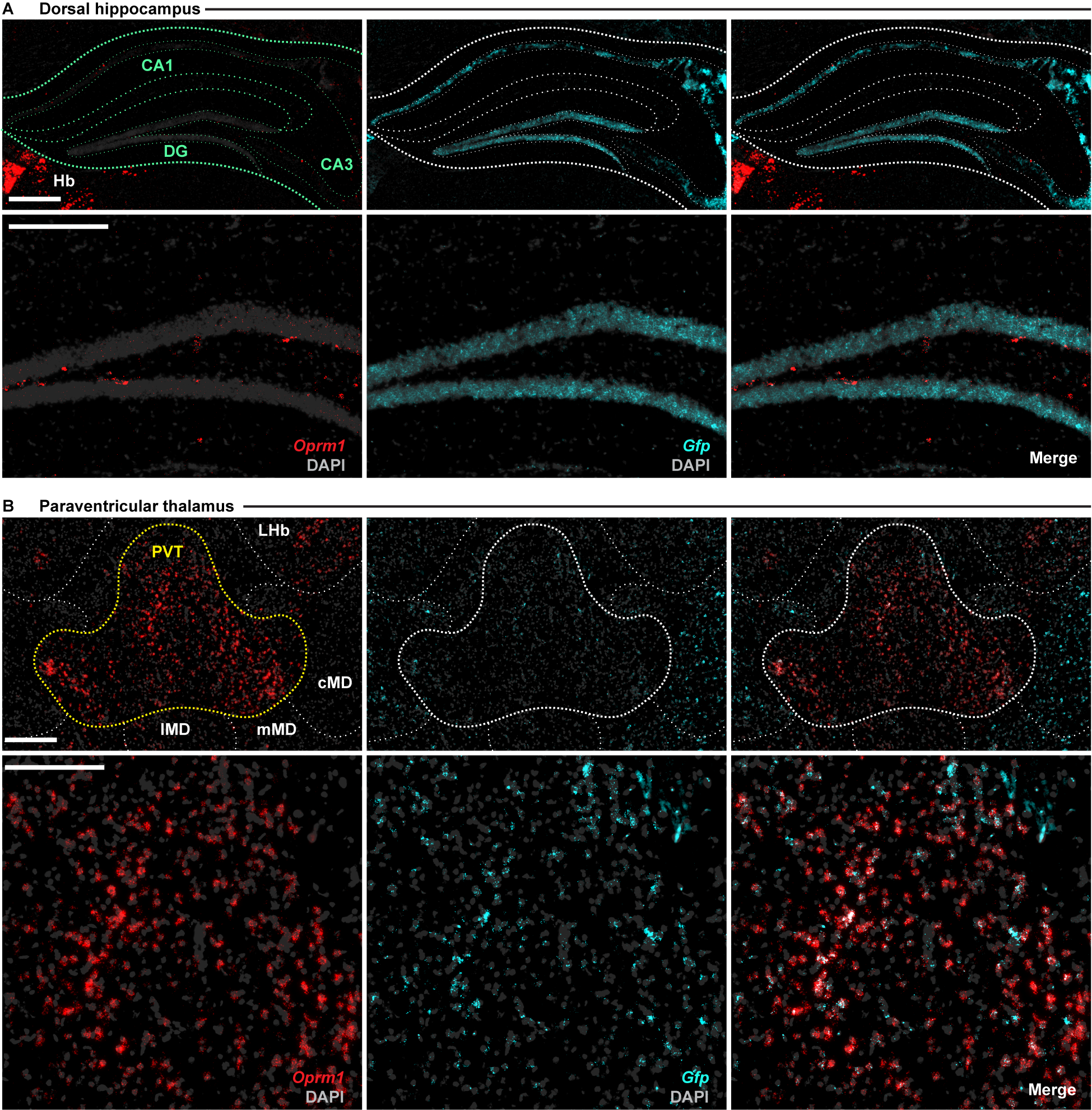

Supplement: S1 Fig — Overlap of Oprm1 (red) and GFP (Cyan) mRNA in (A) the dorsal hippocampus and (B) paraventricular nucleus of the thalamus. Left images: Oprm1 signal (red) and DAPI (grey); Middle: GFP signal (cyan) + DAPI (grey); Right images: Merge of all signals. Top images: Entire ROI with subnuclei indicated; Bottom images: area of densest Oprm1 signal. DG: dentate gyrus; Hb: habenula; CA1: CA1 division of the hippocampus; CA3: CA3 division of the hippocampus; PVT: paraventricular nucleus of the thalamus; LHb: lateral habenula; cMD: Central part of the mediodorsal thalamus; IMD: intermediodorsal nucleus of the thalamus; mMD: medial part of the mediodorsal thalamus. Signal was detected using the RNAScope Multiplex Fluorescence V2 kit. 16μm slices were imaged using the BZ-X800 Viewer software in conjunction with a BZ-X Series automated Keyence microscope. Images were taken at 40x magnification. Mice (n = 2) were homozygous Cre-expressing animals. All scale bars: 200μm. (TIF) [file pone.0270317.s001.tif]

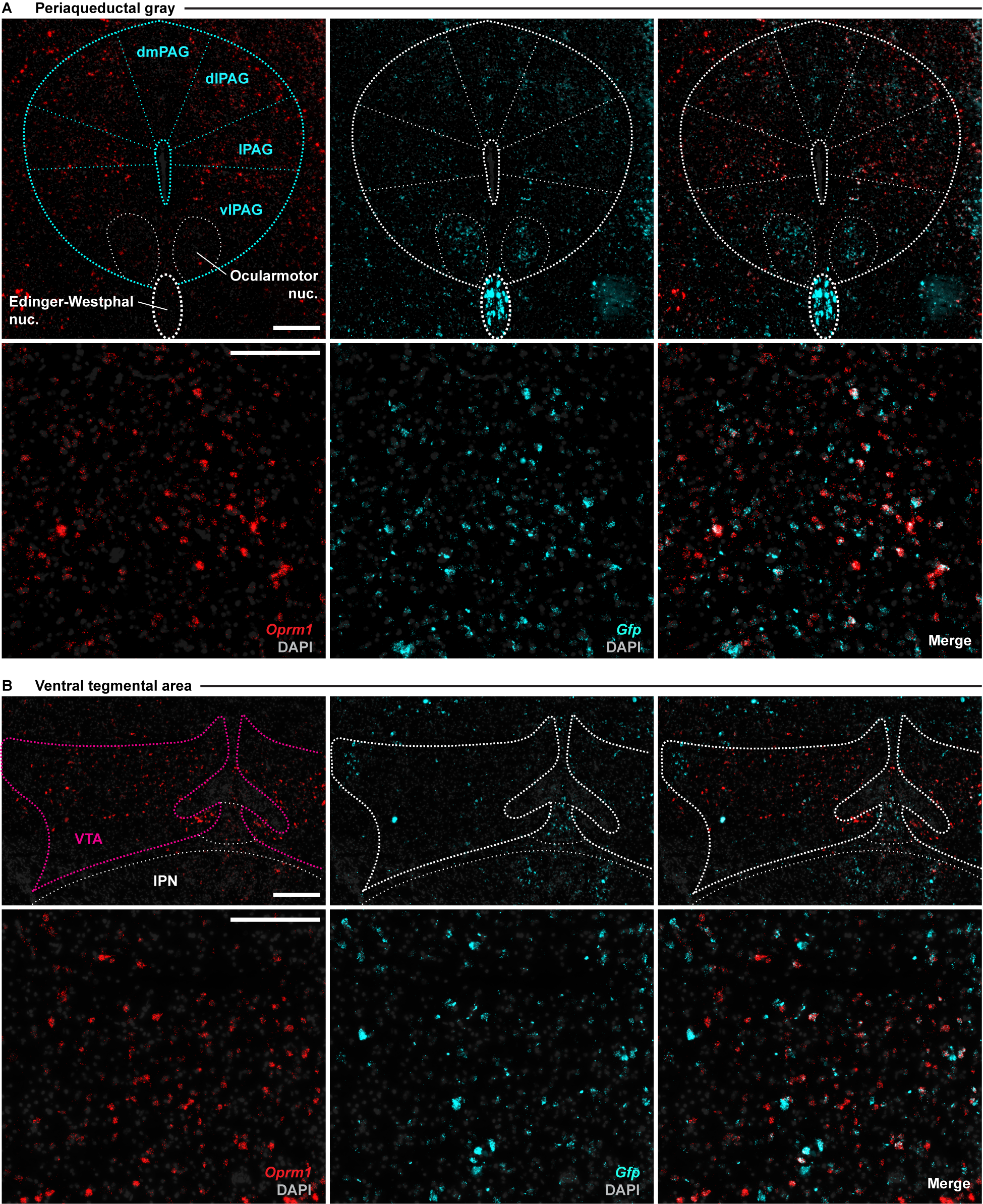

Supplement: S2 Fig — Overlap of Oprm1 (red) and GFP (Cyan) mRNA in (A) the periaqueductal grey and (B) the ventral tegmental area. Left images: Oprm1 signal (red) and DAPI (grey); Middle: GFP signal (cyan) + DAPI (grey); Right images: Merge of all signals. Top images: Entire ROI; bottom images: area of densest Oprm1 signal. dmPAG: dorsomedial Periaqueductal Gray; dlPAG: dorsolateral Periaqueductal Gray; lPAG: lateral Periaqueductal Gray; vlPAG: ventrolateral Periaqueductal Gray; VTA: ventral tegmental area; IPN: intrapeduncular nucleus. Signal was detected using the RNAScope Multiplex Fluorescence V2 kit. 16μm slices were imaged using the BZ-X800 Viewer software in conjunction with a BZ-X Series automated Keyence microscope. Images were taken at 40x magnification. Mice (n = 2) were homozygous for both transgenes. All scale bars: 200μm. (TIF) [file pone.0270317.s002.tif]

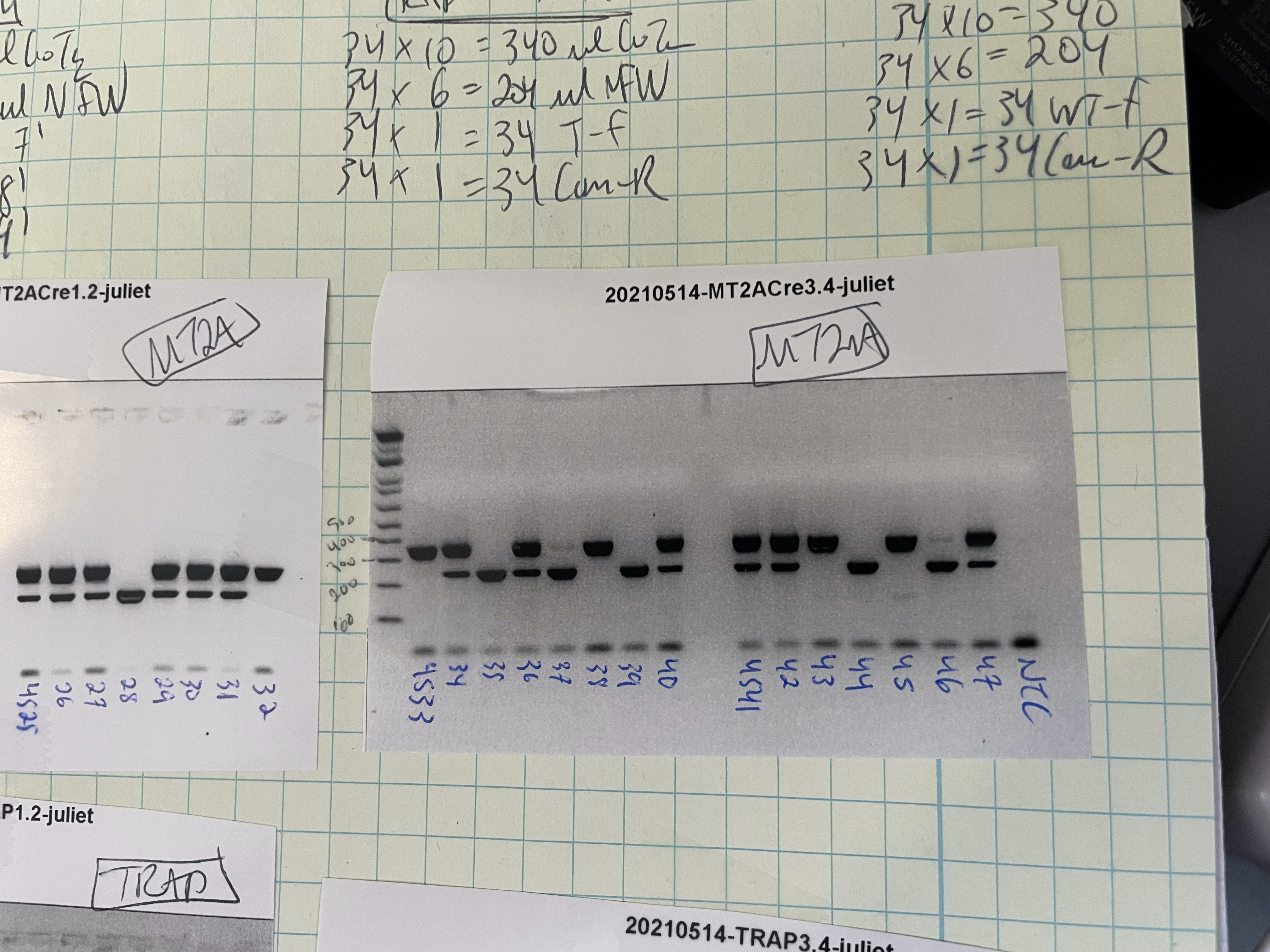

Supplement: S1 Raw image — (JPG) [file pone.0270317.s003.jpg]

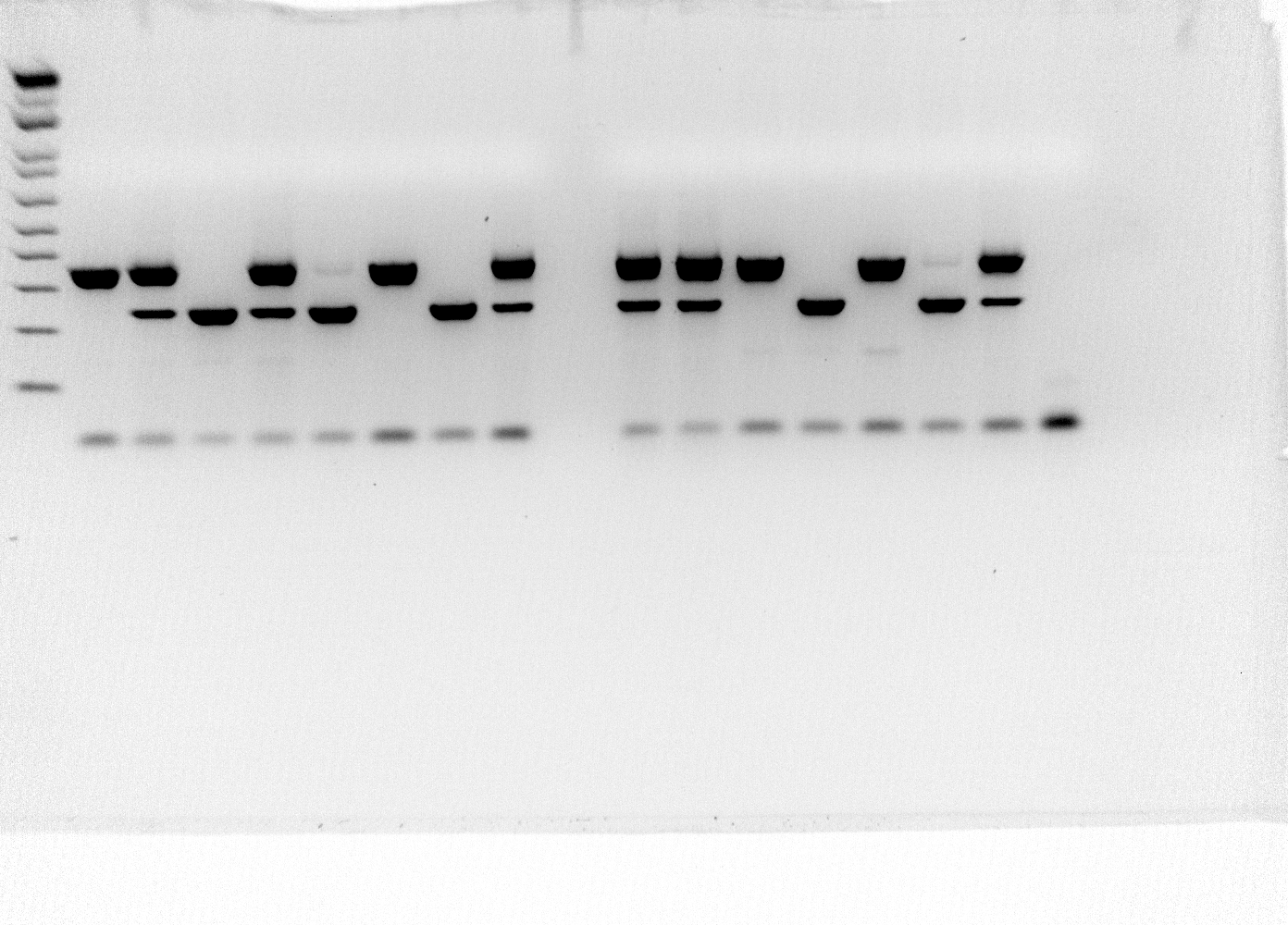

Supplement: S2 Raw image — (PNG) [file pone.0270317.s004.png]
